# Supplementary material for: Dietary Administration of Black Raspberries and Arsenic Exposure: Changes in the Gut Microbiota and Its Functional Metabolites
Source: Metabolites. 2023 Jan 30;13(2):207. doi: 10.3390/metabo13020207 (PMC9967196; doi:10.3390/metabo13020207)
Supplement: Supplementary file 1 [file metabolites-13-00207-s001.zip › metabolites-2107913-supplementary.pdf]

Supplementary Information

Dietary Administration of Black Raspberries and Arsenic Exposure: Changes in the Gut Microbiota and Its Functional Metabolites

Pengcheng Tu [1], Qiong Tang [2], Zhe Mo [1], Huixia Niu [1], Yang Hu [1], Lizhi Wu [1], Zhijian Chen [1], Xiaofeng Wang [1,*] and Bei Gao [3,4,*]

[1] Department of Environmental Health, Zhejiang Provincial Center for Disease Control and Prevention, 3399 Binsheng Road, Hangzhou 310051, China
[2] College of Standardization, China Jiliang University, Hangzhou 310018, China
[3] School of Marine Sciences, Nanjing University of Information Science and Technology, Nanjing 210044, China
[4] Key Laboratory of Hydrometeorological Disaster Mechanism and Warning of Ministry of Water Resources, Nanjing University of Information Science and Technology, Nanjing 210044, China
* Correspondence: xfwang@cdc.zj.cn (X.W.); wintergb2012@gmail.com (B.G.)

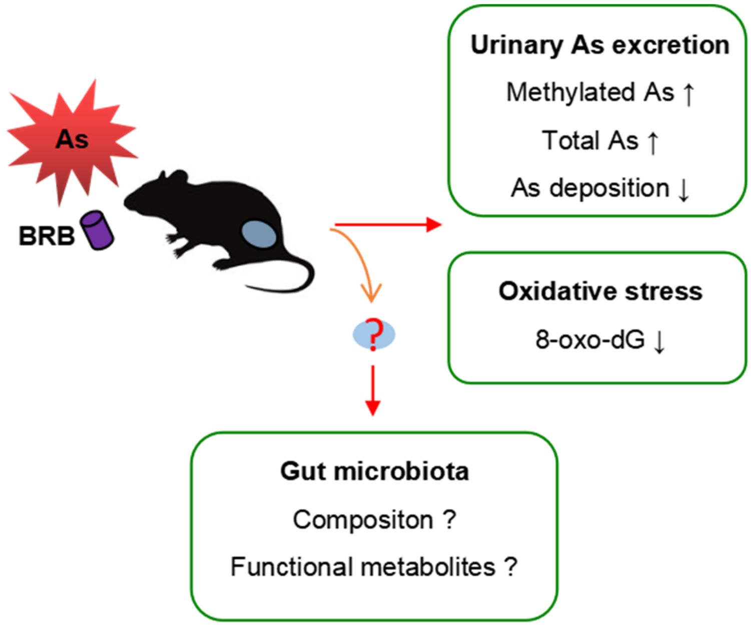

Figure S1. Illustration of effects of BRB consumption on As biotransformation/toxicity.

Order level:

Legend	Taxonomy
	Unassigned;Other;Other;Other
	k__Bacteria;p__Actinobacteria;c__Actinobacteria;o__Actinomycetales
	k__Bacteria;p__Actinobacteria;c__Actinobacteria;o__Bifidobacteriales
	k__Bacteria;p__Actinobacteria;c__Coriobacteriia;o__Coriobacteriales
	k__Bacteria;p__Bacteroidetes;c__Bacteroidia;o__Bacteroidales
	k__Bacteria;p__Cyanobacteria;c__Chloroplast;o__Streptophyta
	k__Bacteria;p__Firmicutes;c__Bacilli;Other
	k__Bacteria;p__Firmicutes;c__Bacilli;o__Bacillales
	k__Bacteria;p__Firmicutes;c__Bacilli;o__Lactobacillales
	k__Bacteria;p__Firmicutes;c__Bacilli;o__Turicibacterales
	k__Bacteria;p__Firmicutes;c__Clostridia;o__Clostridiales
	k__Bacteria;p__Firmicutes;c__Erysipelotrichi;o__Erysipelotrichales
	k__Bacteria;p__Proteobacteria;c__Alphaproteobacteria;o__Caulobacterales
	k__Bacteria;p__Proteobacteria;c__Alphaproteobacteria;o__Rickettsiales
	k__Bacteria;p__Proteobacteria;c__Gammaproteobacteria;o__Enterobacteriales
	k__Bacteria;p__Tenericutes;c__Mollicutes;o__Anaeroplasmatales
	k__Bacteria;p__Tenericutes;c__Mollicutes;o__RF39
	k__Bacteria;p__Verrucomicrobia;c__Verrucomicrobiae;o__Verrucomicrobiales

Family level:

Legend	Taxonomy
	Unassigned;Other;Other;Other;Other
	k__Bacteria;p__Actinobacteria;c__Actinobacteria;o__Actinomycetales;f__Pseudonocardiaceae
	k__Bacteria;p__Actinobacteria;c__Actinobacteria;o__Bifidobacteriales;f__Bifidobacteriaceae
	k__Bacteria;p__Actinobacteria;c__Coriobacteriia;o__Coriobacteriales;f__Coriobacteriaceae
	k__Bacteria;p__Bacteroidetes;c__Bacteroidia;o__Bacteroidales;f__
	k__Bacteria;p__Bacteroidetes;c__Bacteroidia;o__Bacteroidales;f__Bacteroidaceae
	k__Bacteria;p__Bacteroidetes;c__Bacteroidia;o__Bacteroidales;f__Rikenellaceae
	k__Bacteria;p__Bacteroidetes;c__Bacteroidia;o__Bacteroidales;f__S24-7
	k__Bacteria;p__Cyanobacteria;c__Chloroplast;o__Streptophyta;f__
	k__Bacteria;p__Firmicutes;c__Bacilli;Other;Other
	k__Bacteria;p__Firmicutes;c__Bacilli;o__Bacillales;f__Bacillaceae
	k__Bacteria;p__Firmicutes;c__Bacilli;o__Bacillales;f__Paenibacillaceae
	k__Bacteria;p__Firmicutes;c__Bacilli;o__Bacillales;f__Planococcaceae
	k__Bacteria;p__Firmicutes;c__Bacilli;o__Bacillales;f__Staphylococcaceae
	k__Bacteria;p__Firmicutes;c__Bacilli;o__Bacillales;f__Thermoactinomycetaceae
	k__Bacteria;p__Firmicutes;c__Bacilli;o__Lactobacillales;Other
	k__Bacteria;p__Firmicutes;c__Bacilli;o__Lactobacillales;f__Enterococcaceae
	k__Bacteria;p__Firmicutes;c__Bacilli;o__Lactobacillales;f__Lactobacillaceae
	k__Bacteria;p__Firmicutes;c__Bacilli;o__Lactobacillales;f__Streptococcaceae
	k__Bacteria;p__Firmicutes;c__Bacilli;o__Turicibacterales;f__Turicibacteraceae
	k__Bacteria;p__Firmicutes;c__Clostridia;o__Clostridiales;Other
	k__Bacteria;p__Firmicutes;c__Clostridia;o__Clostridiales;f__
	k__Bacteria;p__Firmicutes;c__Clostridia;o__Clostridiales;f__Clostridiaceae
	k__Bacteria;p__Firmicutes;c__Clostridia;o__Clostridiales;f__Dehalobacteriaceae
	k__Bacteria;p__Firmicutes;c__Clostridia;o__Clostridiales;f__Lachnospiraceae
	k__Bacteria;p__Firmicutes;c__Clostridia;o__Clostridiales;f__Peptostreptococcaceae
	k__Bacteria;p__Firmicutes;c__Clostridia;o__Clostridiales;f__Ruminococcaceae
	k__Bacteria;p__Firmicutes;c__Clostridia;o__Clostridiales;f__[Mogibacteriaceae]
	k__Bacteria;p__Firmicutes;c__Erysipelotrichi;o__Erysipelotrichales;f__Erysipelotrichaceae
	k__Bacteria;p__Proteobacteria;c__Alphaproteobacteria;o__Caulobacterales;f__Caulobacteraceae
	k__Bacteria;p__Proteobacteria;c__Alphaproteobacteria;o__Rickettsiales;f__mitochondria
	k__Bacteria;p__Proteobacteria;c__Gammaproteobacteria;o__Enterobacteriales;f__Enterobacteriaceae
	k__Bacteria;p__Tenericutes;c__Mollicutes;o__Anaeroplasmatales;f__Anaeroplasmataceae
	k__Bacteria;p__Tenericutes;c__Mollicutes;o__RF39;f__
	k__Bacteria;p__Verrucomicrobia;c__Verrucomicrobiae;o__Verrucomicrobiales;f__Verrucomicrobiaceae

Figure S2. Legends of bacterial orders or families with each color representing an individual bacterial order or family, respectively.
